# Modeling the drivers of trip satisfaction among Texas coastal anglers using creel survey data

**DOI:** 10.1371/journal.pone.0344688

**Published:** 2026-06-15

**Authors:** Caren Collins, Shambhu Paudel, Elizabeth Harris, Hanna Bauer, Rileigh Hawk, Mark Fisher, Joel Anderson

**Affiliations:** 1 Texas Parks and Wildlife Department, Austin, Texas, United States of America; 2 South Carolina Department of Natural Resources, Charleston, South Carolina, United States of America; National Taiwan Ocean University, TAIWAN

## Abstract

Angler satisfaction can be a complicated concept to explore, because angler perceptions are driven by a wide range of factors that interact in complex ways. Additionally, methods that quantify angler satisfaction often use after-the-fact mail surveys that can be impacted by latent effects such as long recall bias, which can systematically influence angler reporting. Here, we applied boosted regression tree modeling to assess angler satisfaction from creel intercept data collected at boat ramps on the Texas coast from 1991–2023. Anglers returning from fishing were asked to rate their trip on a scale of 0–10, and trip grades were parsed based on anglers targeting three popular sportfish (Spotted Seatrout, Red Drum, and Red Snapper). Angler satisfaction was modeled using observed catch data, weather on the day of trips, and other trip characteristics. Catch characteristics (total catch, angler catch-per-effort, catch length) were the most important predictors of angler satisfaction. There was also temporal and spatial variation in angler satisfaction, with trip grades increasing through time and from north to south. The relative importance of variables driving trip grade were different among angler groups, with weather-related factors (temperature, wind) being slightly more important for offshore Red Snapper anglers. A case study of Red Snapper anglers suggested that increasingly conservative federal and state regulatory measures through the time series appeared to have limited direct effects on angler satisfaction after accounting for temporal trends. Trip-level catch was the main predictor of reported angler trip grades, but spatial and temporal variation in trip grades suggest that angler satisfaction may be local and episodic. Variation in trip grades and the drivers of angler satisfaction among anglers targeting different species underscore the importance of a fisheries-level study design when assessing angler perceptions. These findings shed light on the influences of angler perceptions in Texas coastal fisheries and provide important insights for implementing future regulatory strategies.

## Introduction

Angler satisfaction is a multifaceted psychological value shaped by a variety of factors, including characteristics of the catch itself, environmental conditions and weather on the day of fishing, management regulations that limit catch, and social and cultural interactions [1–3]. The feelings and perceptions formed during and after an angling trip are important for fisheries managers to understand because they reveal what motivates anglers to have a positive or negative experience. High satisfaction levels are often associated with increased compliance with fishing regulations, underscoring the importance of understanding what drives angler satisfaction [4–6]. Conversely an understanding of the necessity for management regulations by anglers can ultimately impact rates of compliance [7,8]. Consequently, awareness of angler satisfaction is an important goal for developing effective management strategies that promote sustainable fisheries and foster positive angler engagement.

It has been widely reported that the overall quality of a fishing trip is often impacted by the quantity and size of the fish caught [5,6]. Catch-related metrics such as catch rate, the average size of landed fish, and total number of fish landed are closely tied to angler satisfaction [6,9] and catch has been shown to be universally valued across a range of species and fishing communities [10–13]. Even when other factors (trip motivation, context and effort) were correlated with angler satisfaction, trip-level catch tended to interact with these motivations to influence overall reported satisfaction [6]. For instance, McCormick and Porter [14] reported that while age was an important driver of angler satisfaction, age was primarily interacting with catch length and catch rates, with younger anglers having more reasonable expectations about the size and number of fish caught. Outcomes such as catch (as opposed to internal factors such as angler motivations), have outsized influence for managing a fishery because they are more easily regulated and are tightly coupled with angler perceptions of the fishery [6].

While tangible catch outcomes are foundational for angler satisfaction, many anglers additionally have other motivations, either environmental or cultural, that may enhance their experience [15,16]. For instance, the desire for environmentally favorable conditions may lead anglers to monitor weather forecasts before and during angling trips, hoping to optimize their chances of success [5,17]. Furthermore, employing fishing guides can significantly elevate the fishing experience, as they provide localized knowledge and assistance tailored to the angler perceived ideal estuarine ecosystems [18,19]. Common perceptions of what constitutes an optimal fishing environment can influence anglers’ planning, with the involvement of knowledgeable guides potentially increasing satisfaction through improved outcomes and a more enjoyable experience [20,21].

In Texas, fishing is a popular activity and an important economic driver, with Texas anglers reporting spending an average of $300 - $600 on angling trips [22]. While several assessments of angler motivations have previously been conducted in Texas [4,12,16,23,24], they have generally targeted either very specific fisheries or angling groups, had narrowly targeted objectives, or have focused primarily in inland or freshwater fisheries. Less emphasis has been placed on assessing the drivers of angler satisfaction among Texas’ coastal anglers. Angler motivations and angler satisfaction are often driven by local factors, and therefore it is important to assess these concepts by understanding local anglers and the species that they are targeting [9]. An assessment of the drivers of angler satisfaction among Texas coastal anglers would be beneficial for fishery managers that are interested in maintaining sustainable fisheries while simultaneously managing angler perceptions. This is particularly true in Texas where anglers are invited to provide feedback on proposed regulatory changes at multiple stages of the regulatory process.

**Fig 1:**
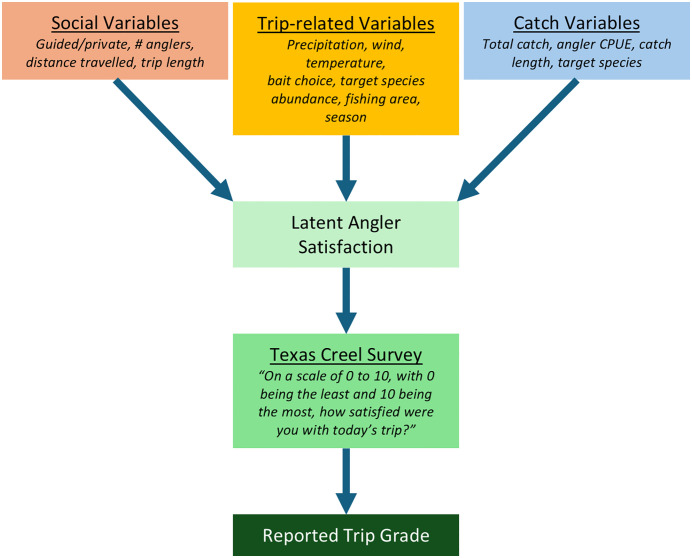
Conceptual model linking social, catch, and trip-related variables on the day of angling trips to a latent angler satisfaction value, which directly informs anglers attitude when reporting trip grade during Texas Parks and Wildlife creel surveys.

The Texas Parks and Wildlife Department (hereafter, “TPWD”) has been collecting post-trip responses from marine recreational anglers through a systematically executed harvest creel program since the 1970’s. The program is designed to obtain estimates of total daylight marine landings, catch per unit of effort, and size composition by species among private and party-boat sport anglers in bays, passes, and the Gulf of Mexico (Gulf of America) waters. Additionally, this program gathers data on environmental parameters and fishing locations and connects this information with catch data for each angling trip. In 1991, two socio-economic questions were incorporated into creel intercepts in order to quantify the species sought and angler satisfaction. This data set is ideal for linking trip-level catch data, environmental conditions, and other trip characteristics to angler satisfaction in distinctive species-specific fisheries, and on the day that trips took place (Fig 1).

The objectives of this work were to (1) summarize spatial and temporal variation in estuarine/marine angler satisfaction in Texas, (2) correlate catch and non-catch related variables to overall angler satisfaction for groups that reported that they were specifically targeting three high-profile recreational fish species (Spotted Seatrout, Red Drum, and Red Snapper), and (3) present a case study of the impact of changing regulations in the Texas Red Snapper fishery on angler satisfaction as reported on the day of angler trips. Assessing angler satisfaction through immediate post-trip interviews has an advantage over previous survey methodologies by eliminating the latent impact of long recall bias inherent in surveys that do not take place on the day of angling trips. The findings reported here will be useful for incorporating the drivers of angler satisfaction into future regulatory decisions in coastal fisheries of Texas, which may subsequently improve angler perceptions of fisheries management and the regulatory process in general.

## Methods

### Data collection

**Fig 2:**
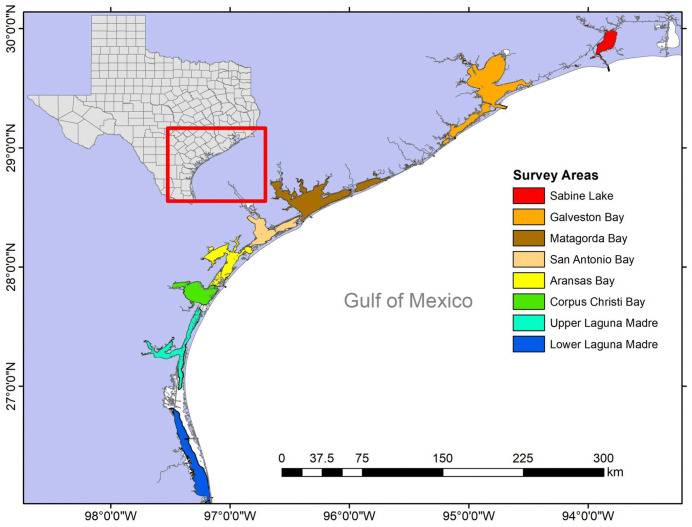
Map of the eight major bay system areas used to stratify boat access angler intercepts in Texas, 1991–2023. All public boater access points in each system were surveyed, and survey days at each ramp were weighted by trailer counts during routine roves (estimated pressure).

The Texas Parks and Wildlife Department has been collecting post-trip data from marine recreational sport anglers through the Texas Marine Sport Harvest Monitoring Program (hereafter “Texas Creel”) since the 1970’s. Texas Creel was designed to obtain estimates of total daylight marine landings, catch per unit of effort, and size composition by species among private and party-boat (guided) sport anglers in bays, passes and nearshore Gulf of Mexico waters. Inventoried boat-access sites are selected in each of eight major bay systems along the Texas Coast (Fig 2). The design is split into two seasons identified as Low-Use (November 21 to May 14) and High-use (May 15 to November 20) based on expected fishing pressure, and site selections for each system are weighted based on the fishing pressure which is estimated using mean roving counts of empty boat trailers.

Interviews of post-trip angling parties were conducted from 10 am to 6 pm on pre-determined weekends and weekdays. While this survey design may exclude angling activity that occurs early in the morning, late in the evening, or along the shore, the program was initially structured this way to maximize the sampled population while remaining efficient with employee time. A previous study suggested that 70% of private boat angling trips occur within the sampled time frame of Texas Creel [25]. Additionally, the mean number of fish observed and measured, mean party size, and mean trip length are greater in the private boat fishery in Texas than in the shore-based fishery [25].

Meteorological data (wind direction, air temperature, sustained wind speed, and presence of fog and rain) is collected on site at both the beginning and end of the survey. All angling parties utilizing the ramp are asked a series of questions regarding trip length, trip type, angler origins (county or state), minor bay of harvest, bait, and landings. If landings are present, interviewers collect up to six lengths (in mm TL) per species and enumerate the remainder of total landings.

A social questionnaire portion was added to Texas Creel in 1987 and shortened in 1991 to include inquiries about trip grade satisfaction (“On a scale of 0 to 10, with 0 being the least and 10 being the most, how satisfied were you with today’s trip?”) and species sought (“Were you fishing for a particular type of fish today?”). In the latter case, responses are transcribed as a numerical value based on a predetermined sportfish code. One angler within the fishing party is selected at random and asked trip grade satisfaction and species sought questions verbatim, ensuring that a group-level response is not obtained. Interviews for the present analysis were aggregated into three groups based on angler responses that were specifically targeting three managed species in Texas: Red Drum, Spotted Seatrout, and Red Snapper, regardless of whether these species were landed. The angler satisfaction score (hereafter “trip grade”) was used as a dependent variable in downstream analyses. Interview data for this study were gathered between 1991–2023 and have been stored continuously by TPWD in a secure relational database. The data for this study were downloaded on October 31, 2024, and are available in the Supplementary information.

Collection of fisheries management data is a statutory requirement of TPWD (TPW Code 66.217, Finfish Research) and angler-intercept data has been collected systematically since 1983 under this mandate. Consent to interview is not verbally solicited, although by the nature of collecting data about public behaviors in a public area (boat ramps), the right of interview refusal is implied and was honored when invoked. No protected or personal sensitive data were collected during interviews. Given these criteria, there is no state requirement for TPWD creel survey activities to be approved by an institutional review board (IRB). All data were downloaded from a curated digital repository between 11/01/2024–11/30/2024.

### Predictor variables

For the purposes of statistical modeling, we examined the influence of a range of predictors on the trip grade variable. Environmental variables included year, bay, wind speed, air temperature, precipitation, and season. All surveys conducted from 1991 through 2023 from all eight major bay systems in Texas were analyzed. Bay was recorded at each inventoried survey site representing Sabine Lake, Galveston Bay, Matagorda Bay, San Antonio Bay, Aransas Bay, Corpus Christi Bay, Upper Laguna Madre, and Lower Laguna Madre. Wind speed and air temperatures were represented as an average of the starting and ending readings for each survey. A binary precipitation variable was generated using combined presence/absence data of rain and fog during the survey. If the presence of rain or fog was noted at the beginning or ending of the survey, then the precipitation variable was recorded as 1. If neither were present, then the precipitation variable was given a 0. The season variable refers to Texas Creel’s delineations between fishing pressure differences observed in low use (generally late fall through early spring) vs high use (late spring through early fall). Creel intercepts are generally lower in low use season due to decreased sampling effort as well as decreased angler effort.

Catch-related predictor variables included the following: total catch, mean length, gill net abundance, area, and angler catch per unit effort (CPUE). Total catch referred to the total number of sought fish landed per interview (e.g., sum of catch of all anglers). Catch length was the mean of all landings (in mm TL) of the targeted species. In two of three cases (Spotted Seatrout and Red Drum), we used estimates of the relative annual abundance of the species targeted as predictors of angler satisfaction. Annual abundance estimates were generated using data from TPWD fisheries independent resource monitoring gill net program. Gill net abundance was calculated using the mean CPUE from fishery-independent gill net catches and was estimated on a coast-wide, annual basis. Gill net abundance was not estimated for Red Snapper; this species occurs primarily offshore in the Gulf of Mexico, and the sparse gill net catches for this species were not an appropriate measure of abundance.

Area fished (federal versus state waters) was only included in Red Snapper models due to the fact that a vast majority of catches of both inshore species (Spotted Seatrout and Red Drum) occur in state waters. In Texas, state waters extend 9 nautical miles from the shore. The Exclusive Economic Zone (EEZ) between 9 and 200 nautical miles marks federal waters. Texas Creel records this data as a numerical code distinguishing harvest between state and federal waters. Angler CPUE was calculated as total landings divided by number of anglers and the trip length in hours (catch/[angler*hour]). Although total catch and angler CPUE were expected to be correlated, there were subtle differences between interpretation of these variables, and it was expected that the modeling approach we applied would be robust to variable multicollinearity.

Additional predictor variables were the number of anglers on board (i.e., party size), trip type, trip length, bait type, and distance traveled. Any person claiming to have not participated in fishing at all during the trip was omitted from the party size variable. Trip type was defined as the type of angling activity for each interview, either private or guided. Trip length (nearest ½ hour) was the elapsed time between when the fishing party left the survey site to the time that they returned and were interviewed. The types of bait used to capture landings were categorized into artificial, dead, live, and other. Artificial was any man-made bait (i.e., lures of varying styles). Dead bait was non-living decapod crustaceans of the Family Penaeidea, non-living finfish, or squid that have been frozen or fresh dead. Live baits are living decapod crustaceans or living finfish. The “other” bait category encompasses any other combination of live, artificial, or dead bait used in an attempt to capture landings. The individual county of residence of all anglers was also recorded for each interview. Texas counties were given a centroid point using ArcGIS Pro geospatial tools based on the county shapefile (Texas Department of Transportation ArcGIS metadata). Each survey site in the Texas Creel program was associated with a specific latitude and longitude. The distance traveled variable was estimated as a straight-line Geodesic distance between the point of origin (county) to the survey site, using ArcGIS online (ESRI, Redlands, CA, USA). Anglers whose residences originated in other states or countries outside the United States were counted as missing data due to the overdispersion of this variable when non-Texas anglers were included.

### Species-specific trip grade analysis

Trip grade was measured as an integer and approximates a Likert scale variable used to measure perception. Applying mean-based parametric statistical models to the full data set was therefore problematic in the sense that a true mean might not be a meaningful representation of trip grade. Therefore, we used ordinal logistic regression to measure trip grade responses to year, species, and year*species. For the sake of simplicity, year was treated as a continuous variable, and species was nominal. The year*species term was included to model interactive effects of these variables. Species-specific annual trip grade analyses were conducted in JMP 18.0 (SAS Institute Inc., Cary, NC USA), with α = 0.05 used to determine whether variables were predictive improvements over the null model. Year and species were plotted against model fitted trip grade *post hoc* to examine the directionality of significant data trends.

### Species-specific multivariate trip grade models

We constructed species-specific prediction models of the trip grade variable using boosted regression tree analysis (BRT). The advantages of BRT for this purpose are numerous; BRT seamlessly models multiple categories of predictor variables, most of the assumptions of traditional statistical tests are relaxed and therefore do not require data transformation, interactive effects that might otherwise require trimming of predictors are handled automatically within the model, and BRT is ideal for handling large, complex data sets [26]. The underlying structure of a BRT is built through producing hundreds or thousands of prediction trees using recursive binary splits, and boosting allows for combination of all trees to create an additive prediction model. In sum, BRT is a recursive ensemble machine learning method, and such approaches may be ideal for making predictions about complex human behavior or perceptions, such as the factors underlying angler satisfaction [27].

Fourteen variables were common to all three species-specific BRT analyses: bay, trip type, trip length, precipitation, air temperature, wind speed, total catch, catch length, number of anglers, angler CPUE, year, season, bait, and distance traveled. For Red Snapper, an additional variable (area – state or federal) was included. For both Red Drum and Spotted Seatrout, gill net abundance of each species was used as a predictor of annual abundance of harvestable individuals. The BRT analyses were conducted using the R library *gbm*, with additional ready-to-use code wraps described in Elith et al. [26]. A BRT analysis requires tuning of three modeling parameters: tree complexity (TC), bag fraction (BF), and learning rate (LR). To evaluate the impact of these modeling parameters on model outcomes, we iteratively built models with a range of TC (4–7), BF (0.5, 0.75) and LR (0.01, 0.05, 0.10) for each species (24 total model parameter combinations for each species). The final model in each case was selected based on the modeling parameters that minimized residual deviance and had at least 1,000 trees, which resulted in slightly different modeling parameters among species (Red Drum: TC = 7, BF = 0.5, LR = 0.01; Spotted Seatrout: TC = 7, BF = 0.75, LR = 0.1; Red Snapper: TC = 7, BF = 0.75, LR = 0.1). Variable importance was estimated using previously established formulae [28,29] where the number of times a variable is selected for branching trees was weighted by the squared improvement to the model, averaged over all trees [26]. For the top 8 predictors in each species-specific model, partial dependence plots were generated with the R package pdp (version 0.8.3) and used to model the marginal effects of predictor variables on trip grade.

Based on observations made during BRT analyses, all three species-specific data sets were combined to examine aggregated trip grade scores compared to two variables: bay and trip type (private/guided). Although these variables were generally found to be minor influences on trip grade in BRT modeling, qualitative examination of GAM plots from the BRT suggested consistency in the relationships of these variables in all three data sets. Mean annual trip grades were compared among groups (bays and trip types) using ordinal regression analysis. In the case of Bay, pairwise comparisons among groups were made *post hoc* using box plots of annualized trip grades in each bay. In the case of Activity, private and guided trip group means were compared directly within the three species target groups.

### Changes in angler satisfaction in the face of regulation change: Red Snapper case study

The Texas creel survey did not directly assess perceptions related to management regulations with targeted survey questions. However, it was our expectation that changes in management regulations might impact angler trip grade. To test this hypothesis, we assessed angler satisfaction in the face of management change indirectly through changes in trip grade scores during different regulatory regimes, with Red Snapper as a special case study.

Northern Red Snapper are the target of perhaps the most controversial recreational fishery in the Gulf of Mexico, due to conflicts and competing interests as well as disagreements over how the stock should be rebuilt following collapse in the 1980s [30]. The fishery has been managed through size limits, bag limits, quotas and season closures, and a variety of other regulations [31]. Although frequent changes in management and season length have been met with frustration in the recreational sector over the past 30 years, previous research has suggested that angler awareness and understanding of the reasoning behind Red Snapper fishery management can improve angler satisfaction in the face of regulation [8]. This finding suggests that angler attitudes about fishing are directly influenced by an understanding of fishery regulations.

Management of the Red Snapper fishery is interjurisdictional and includes state and federal sectors. The state fishery is open year-round and managed through bag limits and a minimum size, both of which have changed over the period examined here. The federal fishery also includes bag and size limits which have been relatively consistent with state regulations, but perhaps the most important element of management in the federal fishery is the opening and closing of the season in each state based on a total allowable catch (TAC) approach.

To examine the impact of fishery regulations on angler trip grade, we computed a mean trip grade for each year of the study for Red Snapper anglers, and modeled annual trip grade using state bag limit, state minimum size and federal season length in each year. First, we conducted univariate tests comparing each predictor to Trip grade. Bag limit and minimum length each changed only twice during the study, so we modeled these as categorical variables using ANOVA, comparing means of the original values to the values of the two updated regulatory periods. Season length varied considerably throughout the length of the study, so in this case we used a general least squares regression approach to model trip grade.

One potential confounding factor related to our univariate analyses is that all three regulatory measures became consistently more restrictive across the time period examined; that is to say, using univariate comparisons made it impossible to determine whether changes in trip grade were directly related to regulations, or alternatively, they were simply related to unmodeled factors associated with changes in perceptions through the sampled period. Stated another way, regulations may simply be marking time; it was observed that angler trip grade increased nearly linearly with year in all three BRT analyses. To account for this, we used an autoregressive integrated moving average analysis (ARIMA) to parse the impacts of year versus regulatory measures on angler satisfaction. An extension of ARIMA is the ARIMAX method, which allows for exogenous factors like fishing regulations to be built into the model as predictors [32]. As a time series model, ARIMAX can measure the impact of predictors on their reported trip grades, while controlling autocorrelation in the time variable (in this case, year). This model is preferred for time series data over traditional statistical models that are naive of temporal autocorrelation. The ARIMAX analysis was conducted using base R functions. The relative importance of predictor variables was assessed using a “leave one out” AIC approach, in which predictors were sequentially removed from the model, and AIC was assessed relative to the full model at each step. The significance of predictors was assessed by standardizing coefficients in the ARIMAX and deriving *z*-scores by dividing standardized coefficients by their standard errors.

## Results

### Species-specific trip grade analysis

**Fig 3:**
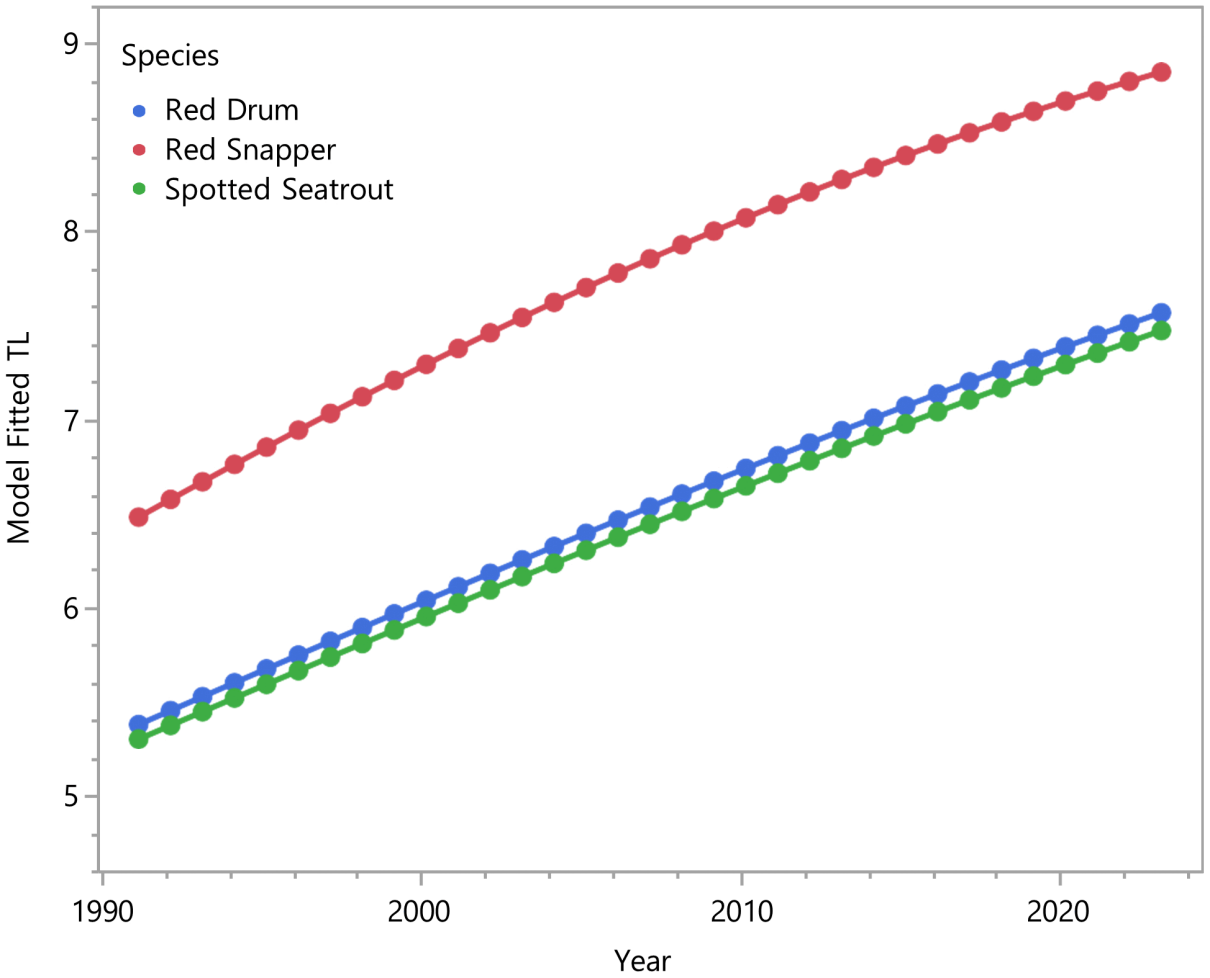
Fitted values of the modeled relationship between year, species, year*species, and trip grade. Reported integer trip grade values were used as dependent variables with year (continuous) and species (categorical) as significant predictors.

When comparing trip grade among species targets and across years, the ordinal logistic regression full model was significant (*χ*^*2*^ = 3474, *d.f.* = 5, *p* < 0.001). Fitted trip satisfaction across the study period was highest in Red Snapper at 8.04, followed by Red Drum at 6.64, and 6.43 for Spotted Seatrout (Fig 3), and the species term was significant (*χ*^*2*^ = 2084, *d.f.* = *2*, *p* < 0.001).

Trip grade also increased through the years for all three species, and this relationship was also significant (*χ*^*2*^ = 3627, *d.f.* = 1, *p* < 0.001). The year*species term was significant, suggesting that the rate of increase in reported trip grades across years was different among the three species. Examination of plots of fitted values of trip grade suggested that although statistically significant, the slopes of trip grade across years did not appear to be qualitatively different from one another.

### Species-specific multivariate trip grade models

**Fig 4:**
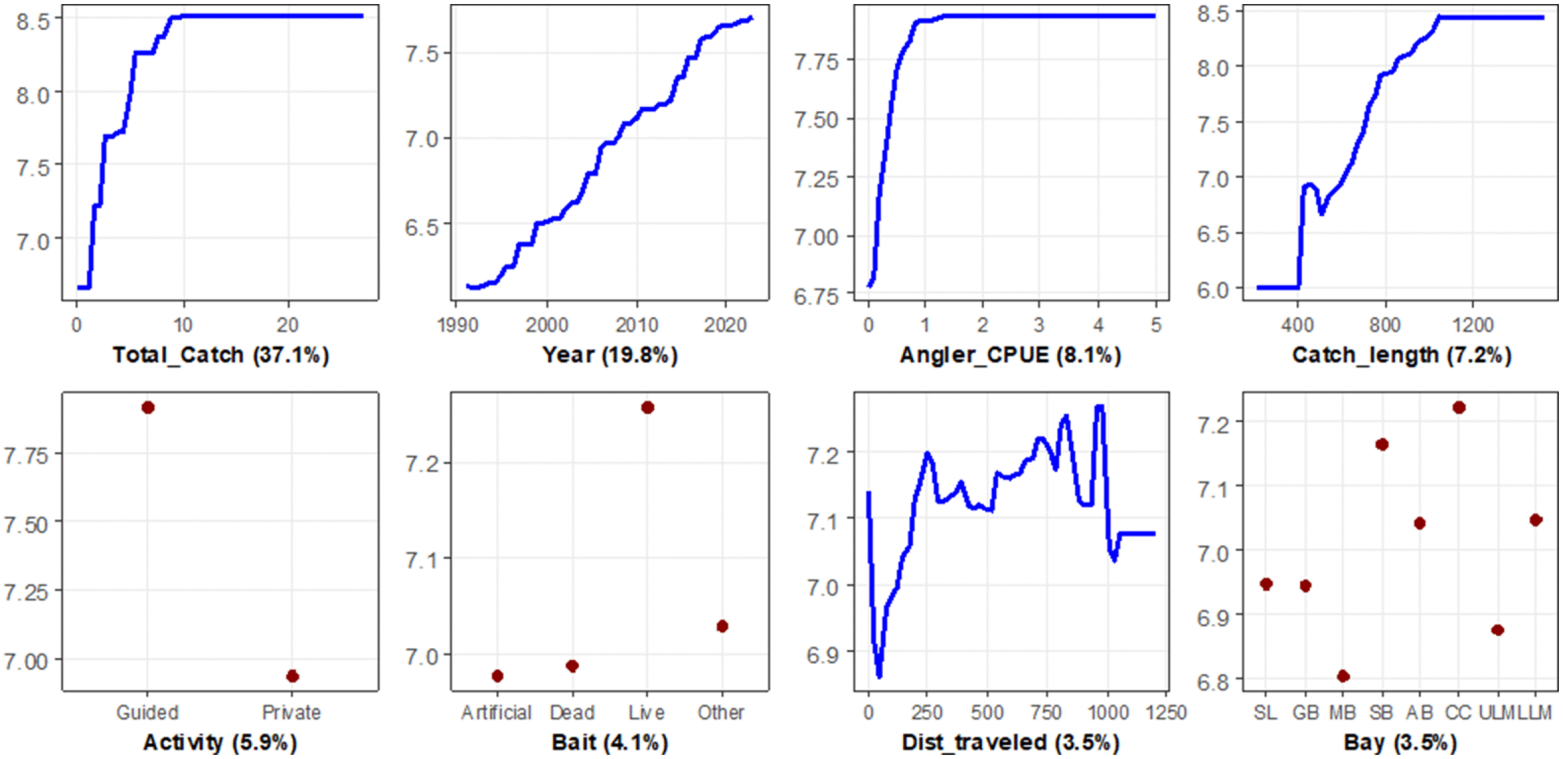
Top 8 variables from a model of trip grade applied to responses from anglers that reported that they were fishing exclusively for Red Drum. The % value in parentheses is the number of trees in a boosted regression tree analysis in which the variable appeared, weighted across all trees, and is used here as a proxy for relative variable influence.

The Red Drum BRT model accounted for approximately 19% of trip grade deviance explained, and total catch was the most important variable predicting trip grade (present in 37% of trees; Fig 4). Higher trip grades were associated with increased overall catch. Year was present in 20% of trees, with trip grades generally increasing steadily through time. Trip grades were also associated with relatively high angler CPUE (8% of trees) and mean catch length (7% of trees). Trip grades generally plateaued at 1 fish/angler, suggesting that trip grade was maximized simply when every angler had contributed to the catch on average. Guided angling trips (trip type, 6% of trees) typically reported higher trip grade than private, non-guided trips. All other variables were present in less than 5% of trees in the Red Drum model.

**Fig 5:**
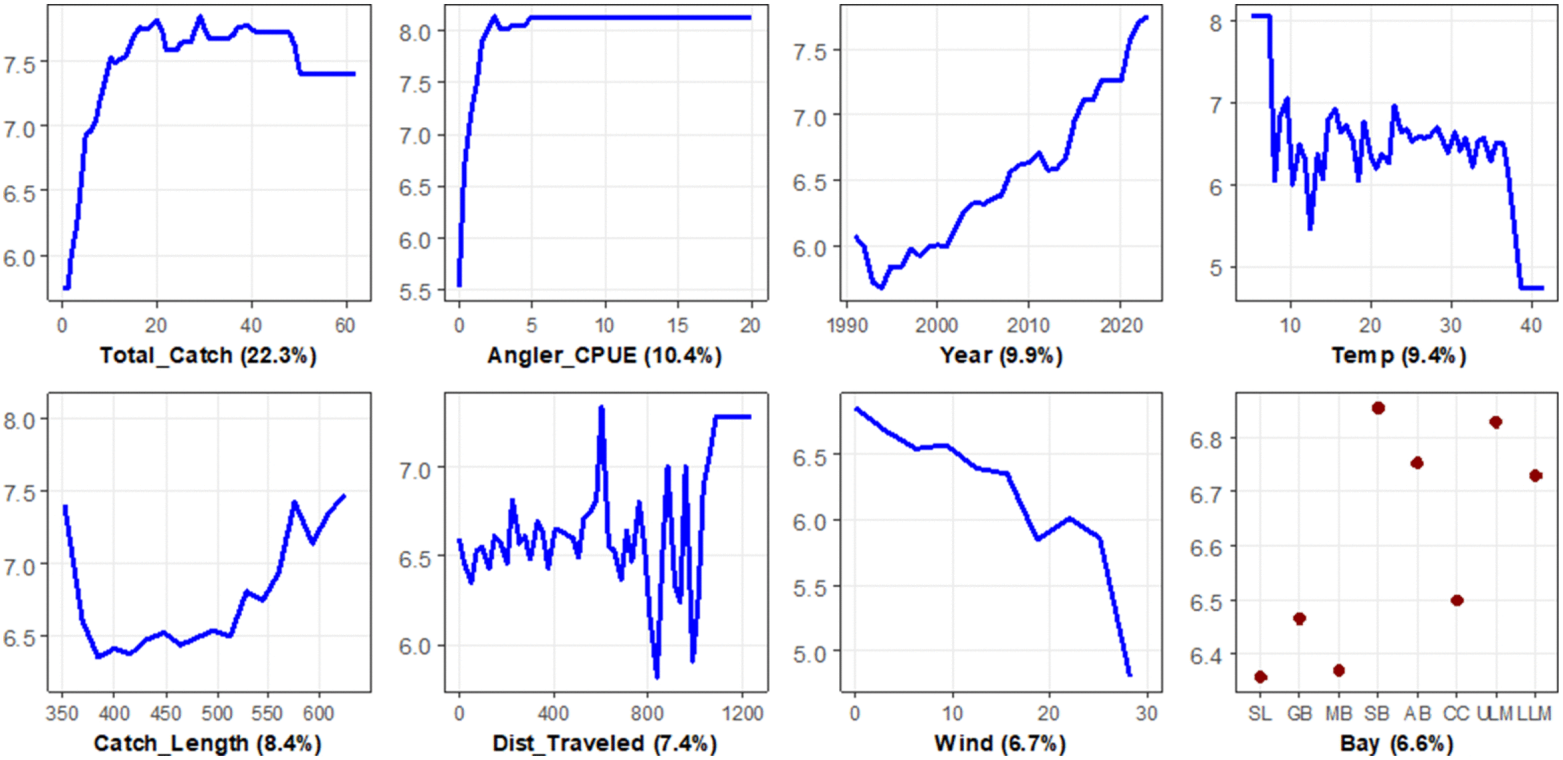
Top 8 variables from a model of trip grade applied to responses from anglers that reported that they were fishing exclusively for Spotted Seatrout. The % value in parentheses is the number of trees in a boosted regression tree analysis in which the variable appeared, weighted across all trees, and is used here as a proxy for relative variable influence.

The Spotted Seatrout BRT accounted for approximately 30% of deviance explained, and once again total catch was the most important variable (21%; Fig 5). Angler CPUE (10%), year (10%), and mean catch length (8%) were all positively correlated with trip grade and had the same general relationships observed in the Red Drum BRT. Air temperature (9%) was important for Spotted Seatrout anglers, with warmer temperatures generally preferred, although trip grade declined at the highest values in this distribution (approaching 40 °C air temperature). Distance traveled (7%) was informative but had a complicated relationship with trip grade that yielded no firm conclusions about its influence. High trip grade was associated with low wind speed (7% of trees), and more southern bays (7%), and was also weakly associated with estimated spotted seatrout abundance (6%) and trip length in hours (5%).

**Fig 6:**
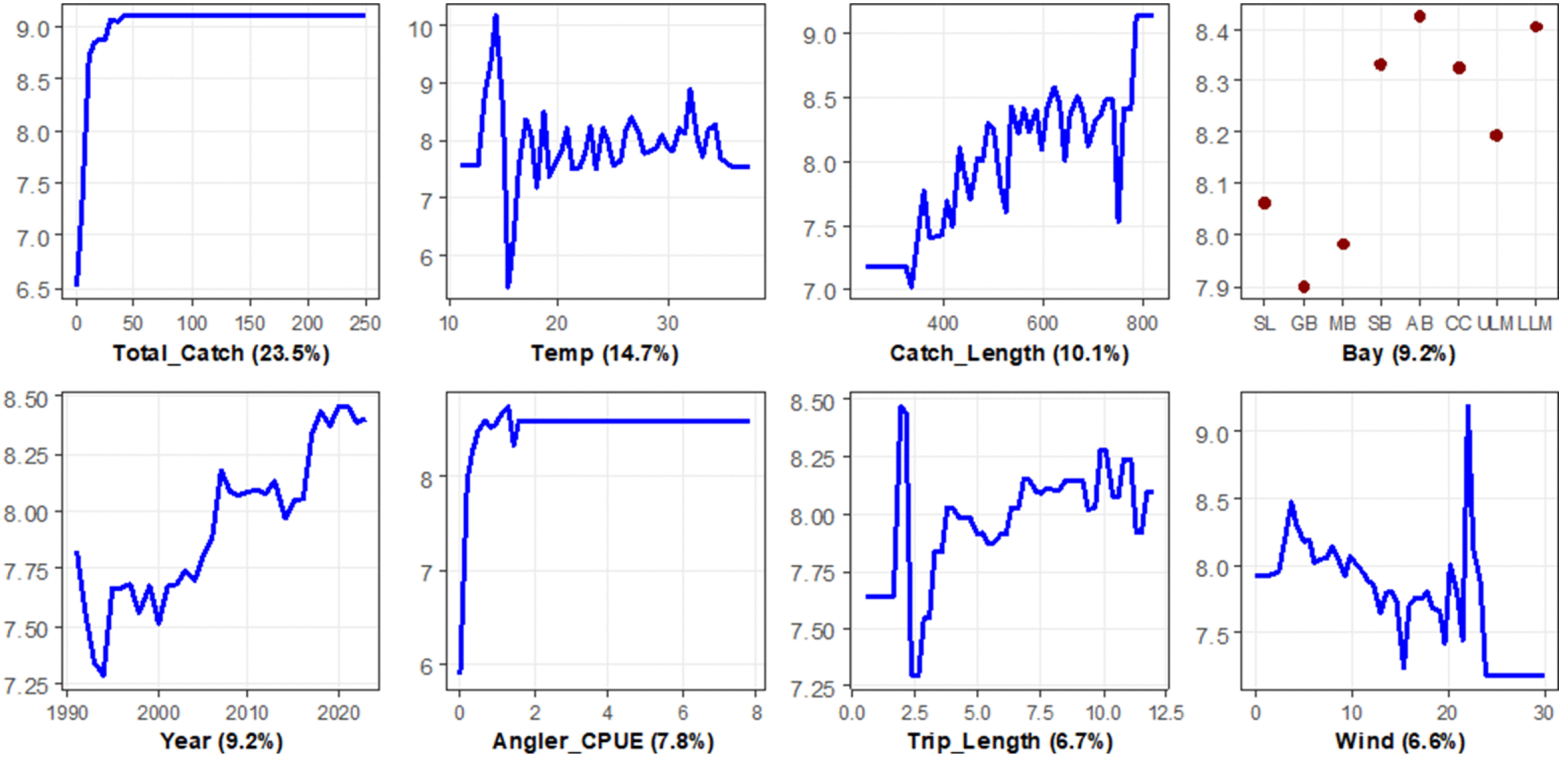
Top 8 variables from a model of trip grade applied to responses from anglers that reported that they were fishing exclusively for Red Snapper. The % value in parentheses is the number of trees a boosted regression tree analysis in which the variable appeared, weighted across all trees, and is used here as a proxy for relative variable influence.

The Red Snapper BRT was highly predictive, accounting for 69% of deviance explained, with total catch as the most important predictor (24% of trees; Fig 6). Air temperature was the 2^nd^ most important variable (15% of trees), although the complexity of this variable compared to fitted trip grade values yielded no strong conclusions; it could be stated that warmer water generally resulted in higher trip grade, although trip grade declined slightly at the highest end of the distribution (approaching 40 °C air temperature). Increased mean catch length (10%), year (9%), and angler CPUE (8%) were all associated with higher trip grades. Trip grades reported in southern bays were generally higher than those in northern bays (bay occurred in 9% of trees), and high trip grades were associated with low wind speeds (7%) and long trip lengths (7%).

**Fig 7:**
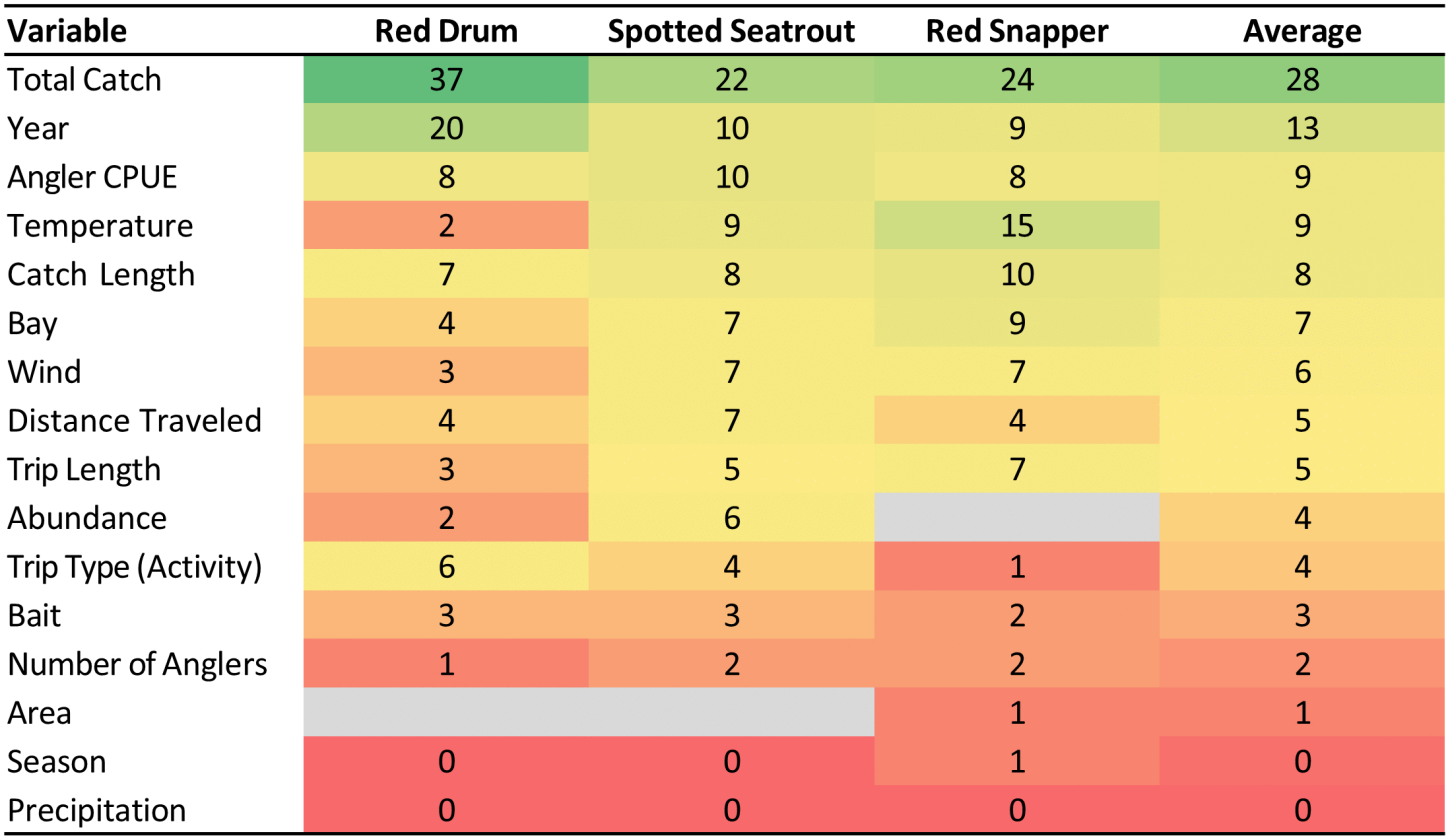
Relative importance of predictor variables in modeling trip grade (i.e., angler satisfaction) as measured during creel interviews for anglers targeting three different managed species, and overall. The numbers in each cell represent the % of trees in which a variable is included in a boosted regression tree (BRT) model of trip grade, weighted across all trees, which is a proxy for variable importance. Cells are colored by importance (green = highly important; red = not important). Gray cells were variables that were not included in all BRT models.

**Fig 8:**
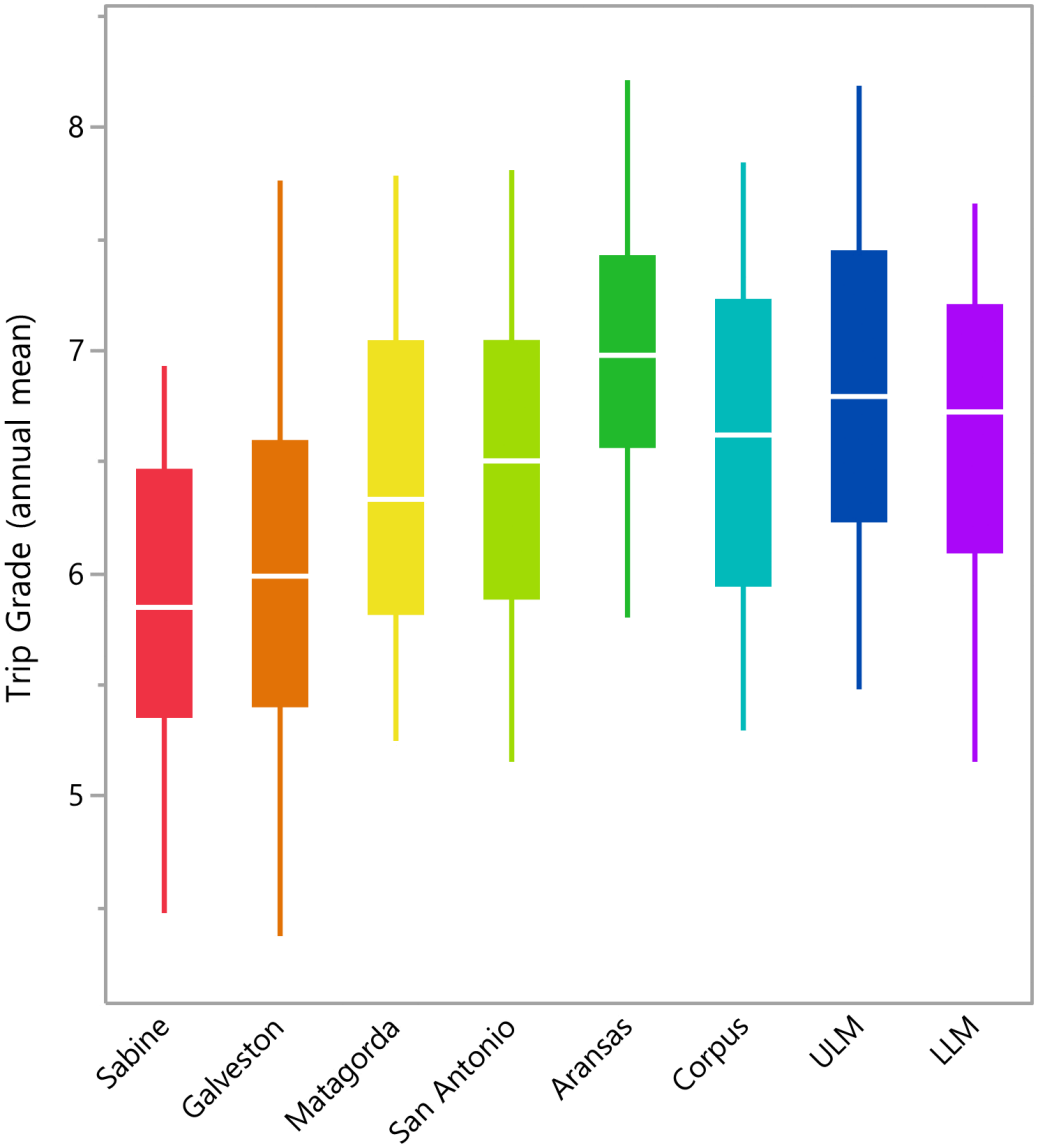
Box plots of mean annual trip grade scores by bay in Texas. Letters on the top of each box plot are indicative of Tukey-Kramer HSD results comparing all means. Letters in common represent comparisons that are not significantly different.

**Fig 9:**
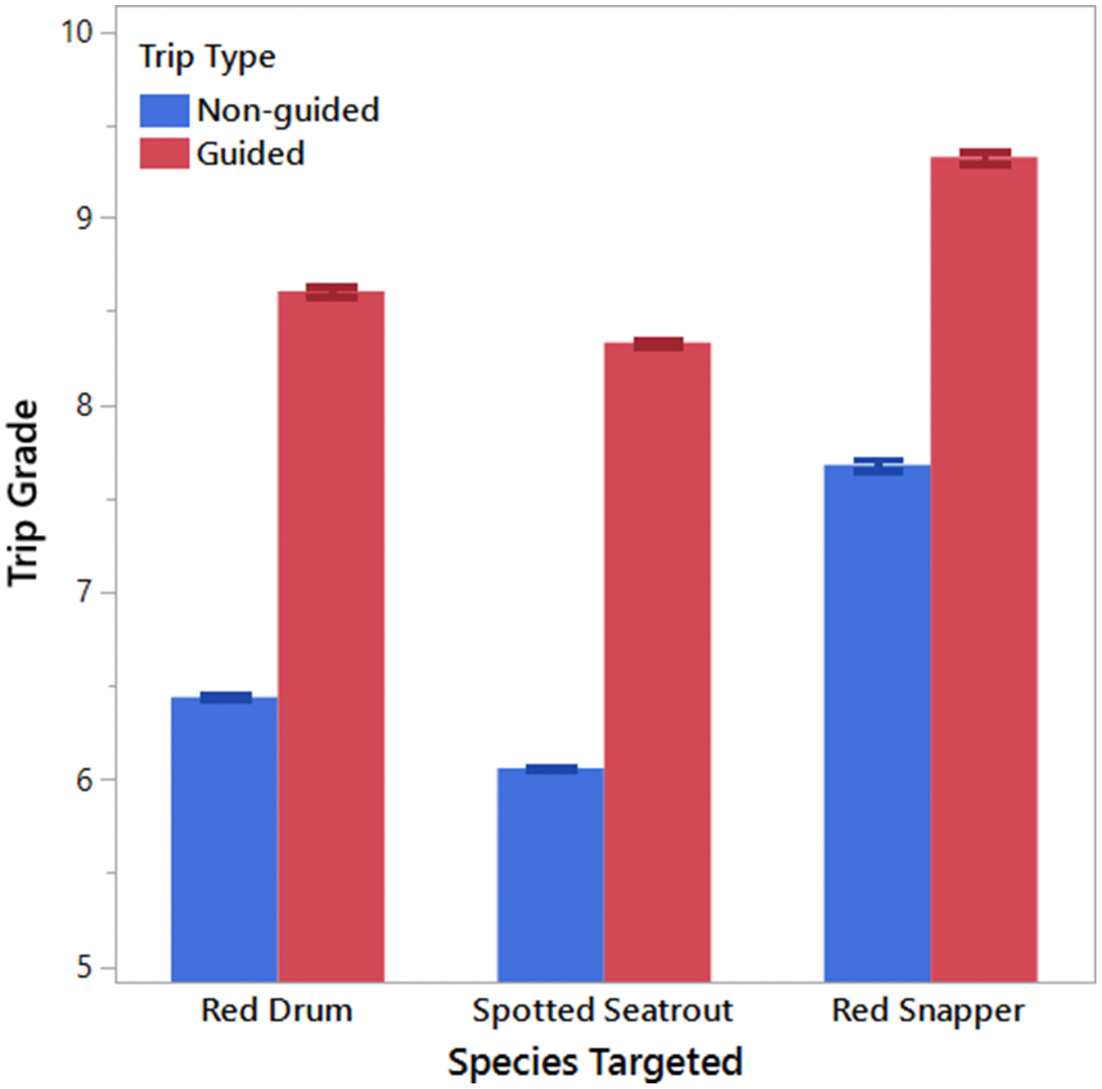
The difference in trip grade between non-guided private trips versus for-hire guided fishing trips. For all three species targets, guided trips had significantly higher mean trip grades. Whiskers at the top of bars represent standard error around the mean value.

Averaged over all models, aspects of the catch itself (total catch, angler CPUE, catch length) were all in the top-6 most important global variables (Fig 7). Year was the 2^nd^ most important global variable, and extrinsic environmental variables were also generally important (temperature ranked 4, wind ranked 7, and bay ranked 6). The ordinal regression analysis of combined data sets suggested that anglers fishing in southern bays in Texas generally reported higher trip grades (*L-R χ*^*2*^ = 1971, *d.f.* = 7, *p* < 0.001; Fig 8). Additionally, guided trips (mean trip grade = 8.2) generally achieved higher trip grades than private, non-guided trips (mean trip grade = 6.2), and the difference was significant (*L-R χ*^*2*^ = 10467, *d.f.* = 1, *p* < 0.001) and consistent across all three species (Fig 9).

### Changes in angler satisfaction in the face of regulation change: Red Snapper case study

Red Snapper bag limits in the state fishery decreased throughout the time period examined, with unlimited catch in 1990, followed by a 7 fish bag limit in 1991, a 5 fish limit in 1995, and a 4 fish limit that started in 1999 and continued to the end of the study. Similarly, the minimum size of allowable harvest became more restrictive, starting at 330 mm (13”) in 1990, 356 mm (14”) in 1994, and 381 mm in 1995 (15”). Season length in the federal fishery was variable throughout, although it started as a year-round fishery in 1990, declined across the time series to 3 days in 2017, and has lasted between 63–93 days in the years since.

**Fig 10:**
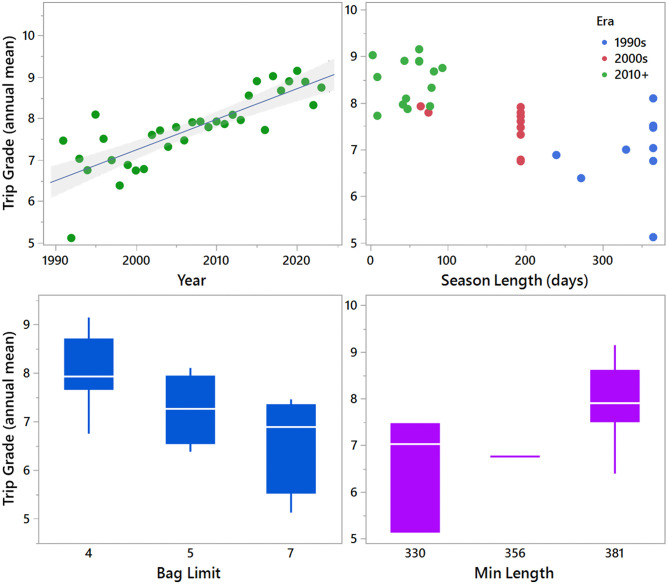
Univariate plots of the correlation of year and changes in regulatory measures to trip grade as reported by anglers targeting Red Snapper in Texas. Season length is the federally mandated number of days in a season as controlled by monitoring the Texas proportion of the Gulf-wide total allowable catch (TAC). Bag limit and minimum length (mm) were based on state regulations, although these were qualitatively consistent with the same regulations in the federal fishery through time. In all cases, trip grade increased with increasingly restrictive fishing regulations, although the results from an analysis of all variables suggested that the influence of Year abated the influence of any regulations.

The ANOVA of bag limit was significant (*F*_*2,30*_ = 7.6, *p* = 0.002), and comparison of means suggested that trip grade was higher for successively more restrictive bag limits (4 fish bag = 8.0 trip grade; 5-fish bag = 7.2; 7 fish bag = 6.6; Fig 10). The ANOVA of minimum size was also significant (*F*_*2,30*_ = 5.0, *p* = 0.012), and once again suggested that trip grade was higher for successively more restrictive minimum lengths (min 330 mm = 6.5 trip grade; 356 mm = 6.7; 381 mm = 8.0). Finally, the least squares analysis of season length suggested that shorter seasons resulted in higher trip grades (*F*_*1,31*_ = 30.5, *r*^*2*^ = 0.480, *p* < 0.001).

The full ARIMAX used to model trip grade in the Red Snapper fishery compared to fishing regulations had an AIC score of AIC = 66.3. Removal of predictors sequentially indicated that the model was considerably worse following removal of the Year term (AIC = 79.7), followed by minimum length (AIC = 67.3) and bag size (AIC = 66.6). The model with season length removed was better than the full model (AIC = 64.3) due to removal of a relatively uninformative term. Comparison of standardized coefficients suggested that only Year and minimum length were significant (Table 1).

**Table 1:** Results of ARIMAX analysis of Red Snapper Trip Grade predicted by Year and regulations, with standardized coefficients (Coef), standard errors (SE) and z-scores derived by dividing absolute value of coefficients by standard error. Significant predictors of Trip Grade had p < 0.05.

Term	*Coef*	*SE*	*z*	*P (higher z)*
Temporal autocorrelation	−0.924	0.137	−6.743	< 0.001
Short-term shocks	0.653	0.246	2.649	0.008
Intercept	−0.013	0.077	−0.170	0.865
Year	0.929	0.189	4.917	< 0.001
Minimum Length	0.463	0.234	1.978	0.048
Bag Length	0.470	0.292	1.608	0.108
Season Length	0.025	0.230	0.111	0.912

## Discussion

### Drivers of angler satisfaction

Perhaps not surprisingly, satisfaction among Texas’ coastal anglers was largely driven by aspects of the catch itself, more than other factors associated with angling trips. The most important variable in the three individual species target models was total catch, which was an enumeration of all fish landed by each interviewed party during the trip. Trip grade increased steadily as total catch increased. Angler CPUE (number of fish caught per angler/hour) was also a top-5 variable in all three species, and a top-3 variable in both of the inshore species (Red Drum and Spotted Seatrout). Interestingly, trip grade increased as CPUE increased from 0 to 1, but generally plateaued thereafter for all three species, suggesting that this variable was simply measuring the increase in angler satisfaction associated with landing at least one fish. Previous studies have similarly reported that catching at least one fish may be a more important element in driving angler satisfaction than even catching a daily limit [33,34].

Catch length (mean length of all harvested fishes) was also a top-5 variable for all species targets, with trip grade increasing steadily as catch length increased. Previous studies have noted that catch-related variables (e.g., total catch, CPUE, catch length) tend to drive angler satisfaction in fisheries world-wide [5–7,9,14], and the results of the current study tend to validate that finding for Texas’ coastal anglers. It is reasonable to assume that the three populations of anglers interviewed in this work, targeting three different coastal species, may have slightly different motivations for fishing, as well as perceptions of trip grade/satisfaction. But in general, all anglers tended to value relatively large catches and catch lengths. This is consistent with at least one previous study of freshwater anglers in Texas where it was reported that angler satisfaction was tightly correlated with catch number and size [12], and collectively these studies suggest that satisfaction among Texas’ anglers is largely tied to angling success in both the inland and coastal fishing sectors.

Year was among the top four most important variables in all three species-specific trip grade models, and a top-3 importance variable for both inshore species. In all three cases, trip grade increased steadily through the years observed in this study, suggesting the presence of a latent variable structure that has improved angler perceptions through time. This finding validates a previous finding that trip satisfaction for fresh and saltwater anglers increased in Texas from 1989–2004 [24] and suggests that this trend has continued. Some of the observed changes in angler trip perception might be driven by changes to angler demographics in Texas over the same time period, and changes in motivations for participating in fishing. For instance, Lu et al. [35] reported that anglers in Texas were increasingly motivated by social factors and a desire to be in a natural environment, and that anglers that were motivated by these factors were more likely to be satisfied with their fishing experience. Additional studies in Texas [16] and elsewhere [2,17] have found similar motivations and may point to drivers of this annual trend.

Anglers that are less catch motivated, including new anglers, tend to rate satisfaction higher than anglers that cite other motivations for fishing [17]. Over the course of the observed period in this study, fishing licenses in Texas increased steadily, including a large influx of new anglers associated with the COVID-19 pandemic. Granneman et al. [16] characterized COVID-19 anglers and found that newer participants comprised a wholly different demographic from retained anglers, and that these anglers tended to assign lower value to catch characteristics (i.e., a “consumptive orientation”) and greater value to the aesthetic experience. Furthermore, Granneman et al. [16] suggested that new COVID-19 anglers were generally indistinguishable from new anglers in other years, and therefore these trends (diversified fishery participants, changes in angler motivations) could reasonably be expected to continue. In this context, the interpretation of the importance of “year” in our modeling may simply reflect increasingly complex angler demographics, paired with changing motivations over time from more catch-oriented anglers to those that are more likely to assign value to the social and aesthetic qualities of fishing experiences. Admittedly, this is a speculative interpretation, as angler demographics and social/aesthetic values were not directly assessed here. Additionally, one caveat to our interpretation of the year trend is that, over the observed time period, the rate of interview refusal (instances where anglers decline to participate in the survey) has also increased. It is conceivable that anglers that have previously rated their experience poorly are more often simply refusing to interview (although this can’t be reliably tested with the current data).

Another interesting aspect of the BRT outputs was the impact of the bay system variable on trip grade, suggesting a geographic component to angler satisfaction. A spatial pattern emerged in which trip grade generally increased from the northernmost bay system (Sabine Lake) to the southernmost bay system (Lower Laguna Madre). Although mean trip grade fluctuated among the targeted analyzed species, the spatial trend was consistent among all three. This trend can possibly be attributed to several factors, including but not limited to 1) differences in habitats and aesthetic quality of the landscape, and 2) demographic diversity among angling communities and 3) differences in localized fishing cultures. Teasing apart these potential drivers of spatial trends in angler satisfaction could benefit fisheries managers in developing an understanding of harvest influences and could aid in developing adaptive management strategies on a regional scale [3].

Regarding habitat, the estuaries along the Texas coast are very diverse in terms of habitat type and abundance, acreage, hydrology, bathymetry, and clarity [36]. For anglers, these differences may manifest as a perception of which areas have the highest abundance or most preferred habitat for their targeted species. Certain types of habitats (e.g., oysters or seagrass) are perceived to provide areas of high abundance for sought after species such as Spotted Seatrout and Red Drum. In Texas, southern estuaries generally have higher salinities, shallower waters, enhanced water clarity, and a greater abundance and diversity of seagrasses [37,38]. These factors contribute to the highest predicted occurrence of juvenile Spotted Seatrout [39] and also support older adult populations of the species [40]. These factors have collectively contributed to the perception of a renowned sport fishery targeting “trophy” fish [41]. More directly related to habitat, previous studies have shown that fishing “context” variables such as natural aesthetics can drive angler satisfaction regardless of whether landings are present [42]. The increased presence of clear and shallow water and abundant seagrasses in Texas’ southern estuaries may directly drive relatively positive experiences compared to the deep and turbid waters and relative lack of seagrasses that characterize northern estuaries.

Regarding demographics and culture, middle and lower coast estuaries in Texas tend to attract anglers from multiple counties across Texas, while the upper coast estuaries (particularly Sabine Lake and Galveston Bay) tend to attract anglers from local and neighboring coastal counties. These angling communities may employ different fishing strategies and have different internal measures of success. For instance, Green et al. [43] found that middle and lower coast anglers in Texas are more likely to hire guided trips than the coast-wide average, and in the current study, guided trips led to higher trip grades than non-guided private trips. Access to fishing sites, overcrowding, and coastal development have also been previously indicated as contributing to angler satisfaction [3,5,12]. These variables all tend to favor higher perceptions of quality for lower coast estuaries which are generally adjacent to more sparsely populated, undeveloped areas. Nevertheless, previous attempts to highlight the cultural differences among Texas’ coastal anglers found that even within very specific angler demographic groups, satisfaction may be driven by complex combinations of motivations at the individual level [1]. While our finding of a clear spatial trend in angler satisfaction may be a clue to the cultural differences among Texas’ coastal angling communities, our data are insufficient to interpret this trend conclusively.

Guided trips were generally rated higher than private trips, although this variable was weighted as relatively low importance in all three species models (10^th^ overall), a result that was likely driven by low predictive power due to the rarity of guided trips (across all three species targets, guided trips made up ~14% of interviews). However, the effect was consistent and robust among species targets, suggesting that guided trips generally result in higher trip grades and anglers that employ for-hire guides typically feel higher trip satisfaction. Guided angling trips generally have higher catch and harvest rates [44] and may result in catching bag limits more quickly. Previous research has suggested that short trips may correspond to higher satisfaction [6], although trip length was only moderately important in the current study. Another interpretation that has been presented in the literature is that anglers may perceive fishing guides as role models, with most anglers responding that guides were “worth emulating” [19]. Smith et al. [20,21] found that inshore recreational fishing guides in Texas were a heterogeneous group that offered highly specialized experiences. The ability to offer specialized experience and localized expertise has likely resulted in a level of satisfaction and value for guided trips that motivates anglers to give higher trip ratings. This relationship between fishing guides and their clients also clearly implies that one strategy for fishery managers in Texas attempting to obtain cooperative buy-in for new fishery regulations might be to focus scoping efforts on fishing guides, who can have an outsized influence on the perceptions of their clients and the greater fishing community.

### Red Snapper case study: do fishery regulations impact reported trip satisfaction?

One of the foundational assumptions of this work, and the reason for including Red Snapper among the three targeted angling communities, was that the frustration associated with increasingly conservative management regulations in the recreational Red Snapper fishery could reasonably manifest as decreasing angler satisfaction through time. Red Snapper are the target of the most controversial fishery in the Gulf of Mexico [45], due to what is perceived as mismanagement and restrictive regulations [30], as well as conflict between commercial and recreational sectors [31,46]. Decreasing bag limits, increasing size limits, and decreasing season lengths have all occurred in this fishery over the last 30 years, which have resulted in observations of changing angler behavior and effort [47, 48] and the development of a derby fishery in the recreational sector [49]. It would be reasonable to assume that frustration in the recreational sector might translate to lower angler satisfaction through time, or even observable changes in angler behavior [50]. Previous work has demonstrated a link between angler sentiment and management regulations, with less restrictive fisheries being associated with increasing angler sentiment [15].

In contrast, we found limited evidence of linkage between trip grade scores reported by Red Snapper anglers and conservative management regulations. Although there were demonstrative associations with all three regulation types (bag limits, size limits, and season lengths), all three variables demonstrated unexpectedly positive associations between increasingly conservative regulations and trip grade, and only minimum length was significant in a full ARIMAX model in which year was included as a predictor. These findings generally validate those of Abbott et al. [51], who found that head boat owners in the Gulf of Mexico reported no notable change in customer trip satisfaction or demand when presented a scenario where the Red Snapper bag limit was one, indicating a puzzling disconnect between the unpopularity of restrictive regulations versus reported angler trip satisfaction.

An interesting consideration when interpreting this disconnect is the balance between awareness of biological sustainability and angler satisfaction [52]. Scyphers et al. [8] reported that increased angler awareness of the science behind fishery management subsequently increased angler satisfaction. Fisheries in the Gulf of Mexico already have some of the highest management-associated angler satisfaction levels in the country [11], and awareness of the basis for regulations in the Red Snapper fishery might be relatively high given the relative importance and popularity of this fishery compared to others in the area. Thus, frustration with management regulations in the recreational fishery might be balanced against understanding the need for regulations and the goal of rebuilding the stock. In this context, on the day of angling trips Red Snapper anglers might simply revert back to catch-based drivers of angler satisfaction (including maximizing harvest rates and sizes) when they report a trip grade at the end of their experience. Increasing abundance and size of Red Snapper in the western Gulf of Mexico have indeed occurred simultaneously with stock rehabilitation [45,53], and these trends may largely be driving angler satisfaction in Texas, despite the unpopularity of the very management measures that have largely caused these improvements. In any event, these data suggest that angler satisfaction may not be tied significantly to restrictive regulations, suggesting that surveys incorporating perceptions of regulatory decisions and the regulatory process may yield misleading results regarding how regulation changes may ultimately impact angler perception or satisfaction.

## Limitations and conclusions

While the data presented here speak to the impact of both intrinsic and extrinsic drivers of angler satisfaction in Texas coastal recreational fisheries, there were inherent limitations to the methodological design that should be noted. The Texas Creel program surveys anglers at boat ramps in Texas; this design was chosen primarily because it maximizes time and efficiency for creel clerks. Approximately 50% of coastal fishing effort in Texas is expended in the boat-based recreational fishing sector, and the mean number of fish observed and measured, mean party size, and mean trip length are greater in the private boat fishery in Texas than in the shore-based fishery [25]. The Texas Creel program was set up as a management tool meant to estimate landings and effort for stock assessments, and this design specifically addresses those data points. However, the attitudes and perceptions of anglers that land their catch in the early morning, or late in the evening, or who are fishing from shore, were admittedly not captured by this design. It is likely that these “missed” anglers might have different motivations and respond differently to latent drivers of angler satisfaction. Additionally, our assessment included no demographic information, which may also represent significant variation in the drivers of angler satisfaction [16]. Future work should attempt to survey the shore-based sector of the Texas coastal angling population and potentially assess demographic differences in each sector; doing so would provide a more robust understanding of the composite recreational angling sector and would more precisely model the social and demographic complexity that is missing from the current data analysis.

Even given these limitations, these data provide insight for management of coastal species in Texas. Texas invites its citizens to participate in the regulatory process by public scoping meetings and public hearings prior to enacting new fishing regulations. While these meetings often include groups that oppose proposed regulations, the current analysis suggests that overall, anglers are more satisfied with their experience than they were 30 years ago, and this finding was repeated across modeling for three of the most heavily fished coastal species. The data also suggest that catch-related variables are foremost on the minds of anglers when assessing their level of satisfaction, although satisfaction was nearly maximized at an angler CPUE value of 1 for all three species (i.e., when everyone caught one fish). These results collectively speak to the complexity of latent angler satisfaction and suggest that while fisheries managers should continue to enact regulations that balance maximized opportunity versus sustainability, there may be drivers of angler satisfaction that are less accessible to traditional fisheries management measures and that require a more nuanced approach.

## Supporting information

S1 File Supporting Information. (ZIP)
